# Cardiac melanocytes influence atrial reactive oxygen species involved with electrical and structural remodeling in mice

**DOI:** 10.14814/phy2.12559

**Published:** 2015-09-23

**Authors:** Hayoung Hwang, Fang Liu, Nataliya B Petrenko, Jianhe Huang, Kurt J Schillinger, Vickas V Patel

**Affiliations:** 1 Penn Cardiovascular Institute, University of PennsylvaniaPhiladelphia, Pennsylvania

**Keywords:** Afterdepolarizations, atrium, melanocyte-like cells, mouse models, reactive oxygen species, remodeling

## Abstract

Cardiac melanocyte-like cells (CMLCs) contribute to atrial arrhythmias when missing the melanin synthesis enzyme dopachrome tautomerase (Dct). While scavenging reactive oxygen species (ROS) in Dct-null mice partially suppressed atrial arrhythmias, it remains unclear if CMLCs influence atrial ROS and structure or if the electrical response of CMLCs to ROS differs from that of atrial myocytes. This study is designed to determine if CMLCs contribute to overall atrial oxidative stress or structural remodeling, and if ROS affects the electrophysiology of CMLCs differently than atrial myocytes. Immunohistochemical analysis showed higher expression of the oxidative marker 8-hydroxy-2′-deoxyguanosine in Dct-null atria versus Dct-heterozygous (Dct-het) atria. Exposing isolated CMLCs from Dct-het and Dct-null mice to hydrogen peroxide increased superoxide anion more in Dct-null CMLCs. Trichrome staining showed increased fibrosis in Dct-null atria, and treating Dct-null mice with the ROS scavenger Tempol reduced atrial fibrosis. Action potential recordings from atrial myocytes and isolated Dct-het and Dct-null CMLCs in response to hydrogen peroxide showed that the EC_50_ for action potential duration (APD) prolongation of Dct-null CMLCs was 8.2 ± 1.7 μmol/L versus 16.8 ± 2.0 μmol/L for Dct-het CMLCs, 19.9 ± 2.1 μmol/L for Dct-null atrial myocytes, and 20.5 ± 1.9 μmol/L for Dct-het atrial myocytes. However, APD_90_ was longer in CMLCs versus atrial myocytes in response to hydrogen peroxide. Hydrogen peroxide also induced more afterdepolarizations in CMLCs compared to atrial myocytes. These studies suggest that Dct within CMLCs contributes to atrial ROS balance and remodeling. ROS prolongs APD to a greater extent and induces afterdepolarizations more frequently in CMLCs than in atrial myocytes.

## Introduction

Atrial fibrillation (AF) is the most common clinical arrhythmia and afflicts over 3 million individuals in the United States (Naccarelli et al. 2009). We recently identified a population of cardiac melanocyte-like cells (CMLCs), in mice and humans that are electrically excitable and contribute to atrial arrhythmias in response to oxidative stress (Levin et al. 2009). Our initial report suggests mice engineered with germline deletion of the melanin synthesis enzyme dopachrome tautomerase (Dct), which is specifically expressed by CMLCs in the atrium, are more susceptible to atrial arrhythmias than wild-type mice (Levin et al. 2009). In addition, we previously showed that atrial arrhythmia induction is partially suppressed in Dct-null mice by scavenging reactive oxygen species (ROS) in vivo. However, it remains unclear from our previous studies if CMLCs contribute to the overall balance of atrial ROS that influences oxidative stress and remodeling. In addition, it is not known whether Dct deletion just altered the electrical response of CMLCs to ROS, since ROS is also known to influence arrhythmogenicity of the atrial myocardium (Lin et al. 2003).

To address these questions we assessed the effects of ROS on atrial oxidative modification, structural remodeling and the electrophysiological response of isolated CMLCs and myocytes from the atria of Dct-positive and Dct-null mice. The findings from these studies suggest that Dct expressed by CMLCs is a regulator of whole atrial oxidative stress, which contributes to electrical and structural remodeling that promotes atrial arrhythmias in mice.

## Methods and Materials

### Animals

The creation of *DctCre* germline knock-in (Guyonneau et al. 2004) and *Z/EG* transgenic mice (Novak et al. 2000), and the associated primer sets used for distinguishing mutant alleles from wild-type alleles have been previously described (Guyonneau et al. 2004), The mice used in this study were cross-bred five generations or more on a C57BL/6 background. All protocols conformed to the guidelines established by the Association for the Assessment and Accreditation of Laboratory Animal Care and were approved by the University of Pennsylvania Animal Care and Use Committees.

### Atrial myocyte isolation

The protocol we used is similar to those previously published for isolating murine atrial myocytes (Levin et al. 2009; Liu et al. 2008). Briefly, hearts from homozygote and heterozygous *DctCre::Z/EG* littermate mice (aged 3–4 weeks) were excised through a sternotomy following heparinization (100 U i.p.) and euthanasia with CO_2_. The hearts were then mounted on a Langendorf apparatus and perfused with Ca^2+^-free Tyrode’s solution for 6 min at a flow rate of 3.0–3.5 mL/min and a temperature of 36–37°C, followed by 12–15 min of perfusion with Ca^2+^-free Tyrode’s solution containing collagenase (collagenase B + collagenase D, Worthington Biochemical; Lakewood, NJ), plus protease type XIV (0.35 U/mL, Sigma-Aldrich Chemical Co.). When the hearts appeared pale and flaccid they were removed from the Langendorf apparatus, the atria were dissected away and kept in Ca^2+^-free Tyrode’s solution with 1 mg/mL of bovine serum albumin (Fraction IV, Sigma-Aldrich). Sections of atrial tissue were then triturated gently with a Pasteur pipette to dissociate individual myocytes.

### CMLC isolation

Hearts were removed from heterozygous and homozygous *DctCre::Z/EG* mice as previously described (Levin et al. 2009; Hwang et al. 2014). The atria were then dissected away from the atrioventricular annulus under a dissecting microscope, rinsed free of blood in PBS (phosphate buffered saline) and incubated in 0.25% trypsin at 37°C for 30 min. Single cells were isolated from the atria by repeated, gentle pipetting in PBS containing 10% FBS (fetal bovine serum), followed by filtration through a 40-*μ*m nylon mesh (Corning Life Sciences, Tewksbury, MA). The cells were then washed with PBS × 3 and placed in melanocyte isolation media for 6–8 h. Unattached myocardial cells, including myocytes, were rinsed away and patch clamp recordings were obtained from green fluorescent cells the next day. G418 (Invitrogen) at a concentration of 100 *μ*g/mL was added to the culture medium to suppress fibroblast growth.

### Single cell electrophysiological recordings

This protocol is similar to those previously published for recording action potentials (Levin et al. 2009; Kim et al. 2011; Wang et al. 2009; Qiao et al. 2011). Briefly, recordings were obtained from single CMLCs and atrial myocytes isolated from homozygote *DctCre* and littermate heterozygous *DctCre* mice with the *Z/EG* transgene (Guyonneau et al. 2004) at room temperature using the whole-cell patch clamp configuration (Hamill et al. 1981). Acton potentials were elicited by injecting a 0.2–0.4 nA pulse with a 1–2 msec pulse width using an Axopatch 200B amplifier interfaced to a PC-computer via a 12-bit A/D interface running the *pClamp 9.2* software. Traces were digitized at 20-kHz and filtered at 5-kHz prior to storage for off-line analysis. The bath solution contained (in mmol/L): NaCl 132, KCl 4.8, HEPES 10, CaCl_2_ 2, MgCl_2_ 1.2, pH = 7.4 with NaOH. The pipette solution contained (in mmol/L): KCl 110, Na_2_-ATP 5, EGTA 11, HEPES 10, CaCl_2_ 1, MgCl_2_ 1, pH = 7.3 with KOH. Series resistances was compensated electronically (75–90%) resulting in uncompensated voltage errors less than 5 mV. Patch pipettes were fashioned from borosilicate glass and fire-polished to a final resistance of 2.0–2.5 MΩ for atrial myocytes or 3.0–3.5 MΩ for CMLCs.

### Histological and immunohistochemical analysis

Hearts were harvested from wild-type and homozygote *DctCre* mice (aged 8–10 weeks) following heparinization (100 U i.p.) and euthanasia by pentobarbital overdose (50 mg/kg i.p.). The hearts were then rinsed free of blood in PBS, fixed in 4% paraformaldehyde overnight, dehydrated through an ethanol series and embedded in paraffin for sectioning. Sections (5–6 *μ*m) were deparaffinized for Masson’s trichrome staining and immunohistochemical analysis as previously described (Levin et al. 2009; Liu et al. 2008; Ismat et al. 2005). Sections stained with Masson’s trichrome were measured for the fraction of fibrous tissue content by quantitative image analysis using the Image-J software suite (National Institutes of Health) (Liu et al. 2008). For immunohistochemical analysis, tissue sections were deparaffinized and antigen retrieval was performed by heating in a pressure cooker. Sections were then stained with either a mouse monoclonal antibody directed against 8-hydroxy-2′-deoxyguanosine (8-OHdG), a modified DNA base produced during oxidative stress (Cat. No. 24326, OxisResearch International Inc.), or a rabbit polyclonal antibody directed against tyrosinase (Cat. No. sc-15341, Santa Cruz Biotechnology Inc., Santa Cruz, CA). Primary antibodies were detected using either a TexasRed or Alex488 conjugated secondary antibody, respectively (Yamamoto et al. 2003). The fluorescence signal after staining was detected and recorded using a fluorescent microscope equipped with a CCD camera (Eclipse i80, Nikon, Japan). Twenty-six separate images were obtained at 400× from each of three different Dct-Cre and control wild-type atria which covered the entire atrial area from each preparation. The area of fluorescent signal was quantified using the count nuclei application in the Metamorph software package (Version 6.3, Molecular Devices) and compared to the total area of each image, as previously described (Liu et al. 2008).

### Induction and detection of ROS in isolated CMLCs

CMLCs were isolated from homozygous and heterozygous *DctCre::Z/EG* mice and plated as described above. The cells were then rinsed in Tyrode’s solution × 3 and incubated with hydrogen peroxide (5–10 μmol/L, Sigma) in Ca^2+^-free Tyrode’s solution for 4 h. Endogenous superoxide anion was detected by adding 5 μmol/L dihydroethidium (DHE, Invitrogen) to the dish for 30 min and then rinsing the dish with Ca^2+^-free Tyrode’s solution. This was followed by analysis of single, GFP-positive CMLCs on a fluorescent microscope (Nikon 80i) equipped with green and red fluorescence filter sets and a CCD camera. Relative fluorescence intensity was quantified by measuring pixel intensity under red fluorescence within each cell that was also GFP-positive using the Metamorph software suite (version 6.3) as previously described (Ismat et al. 2005). A minimum of three independent samples were analyzed for each treatment condition. The upper 95% of each measurement was pooled and averaged, and the resulting datasets were analyzed for significance.

### Statistical analysis

All values are expressed as the mean ± one standard deviation (SD). Differences between two groups were analyzed using two-tailed Student’s *t* test or, when not normally distributed, using a nonparametric Mann–Whitney *U* test. Differences in means among multiple datasets were analyzed using one- or two-way ANOVA with treatment or genotype as the independent factors. When ANOVA showed significant differences, pairwise comparisons between means were tested using Tukey post hoc analysis. When data were not normally distributed, ANOVA on ranks was used with the Kruskal–Wallis test, followed by pairwise comparison using the Dunn test. Afterdepolarizations were assumed to follow a Poisson distribution and the Kolmogorov–Smirnov test was used to assess statistical differences between the groups for this analysis. Statistical analyses were performed using the SPSS statistical package (Version 20, SPSS Inc., Kacharakanahalli, Bangalore). The EC_50_ of hydrogen peroxide upon action potential duration at 90% repolarization was obtained by fitting the data to a Boltzmann distribution with nonlinear regression analysis using the GraphPad Prism software suite (Version 6.04, GraphPad Software, Inc., La Jolla, CA). A *P* value less than 0.05 was considered significant in all analyses.

## Results

### Dct in CMLCs contributes to atrial myocardial oxidative balance

**Figure 1:**
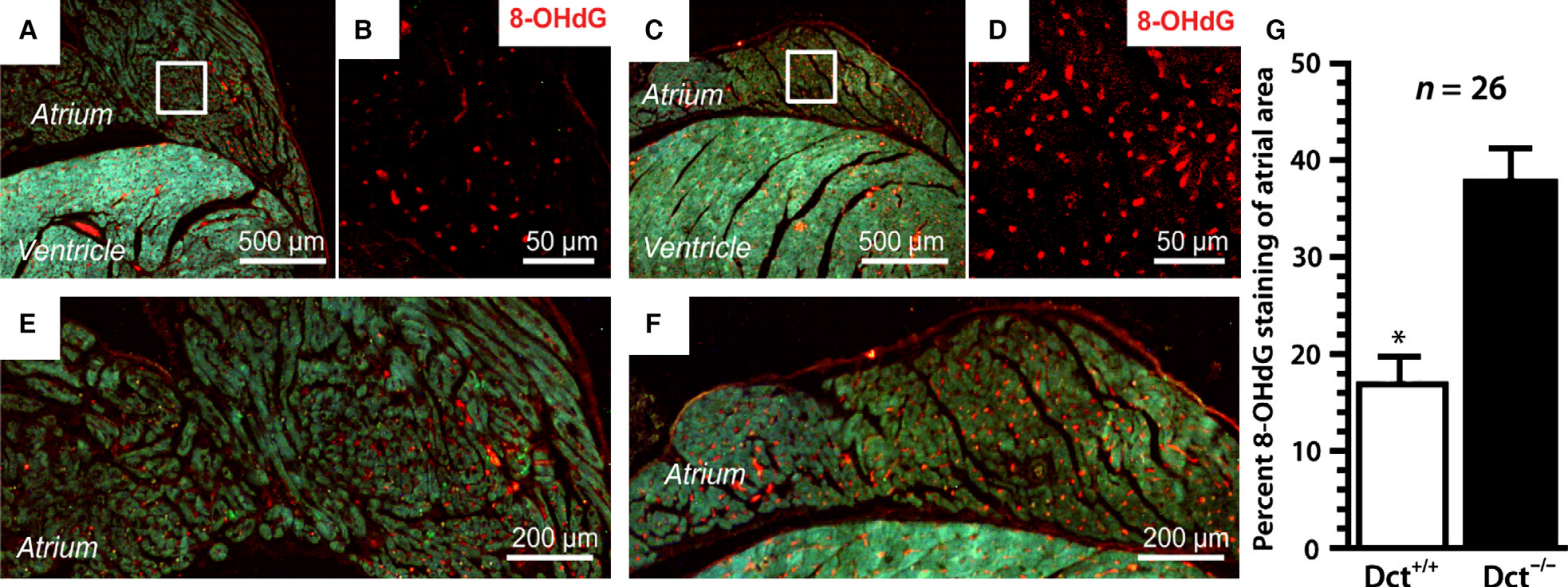
Dct reduces atrial oxidative modifications. Shown are representative hearts from wild-type (A–B and E) and Dct-null (C–D and F) mice following immunohistochemical staining with an antibody against the oxidative marker 8-hydroxy-2′-deoxyguanosine (8-OHdG). (A) and (C) are taken at 40× magnification, (B) and (D) are taken at 400×, whereas (E) and (F) are taken at 100×. The higher power images in (B) and (D are enlargements of the area within the white boxes in (A) and (C), respectively. (E) and (F) were taken using triple filter settings to show gross morphology and anti-8-OHdG staining. In (G) the bar graph compares the mean percentage of anti-8-OHdG staining throughout the atrium between the two groups. At least 26 sections were analyzed from three different hearts in each group. Error bars represent the standard deviation. *P < 0.05.

**Figure 2:**
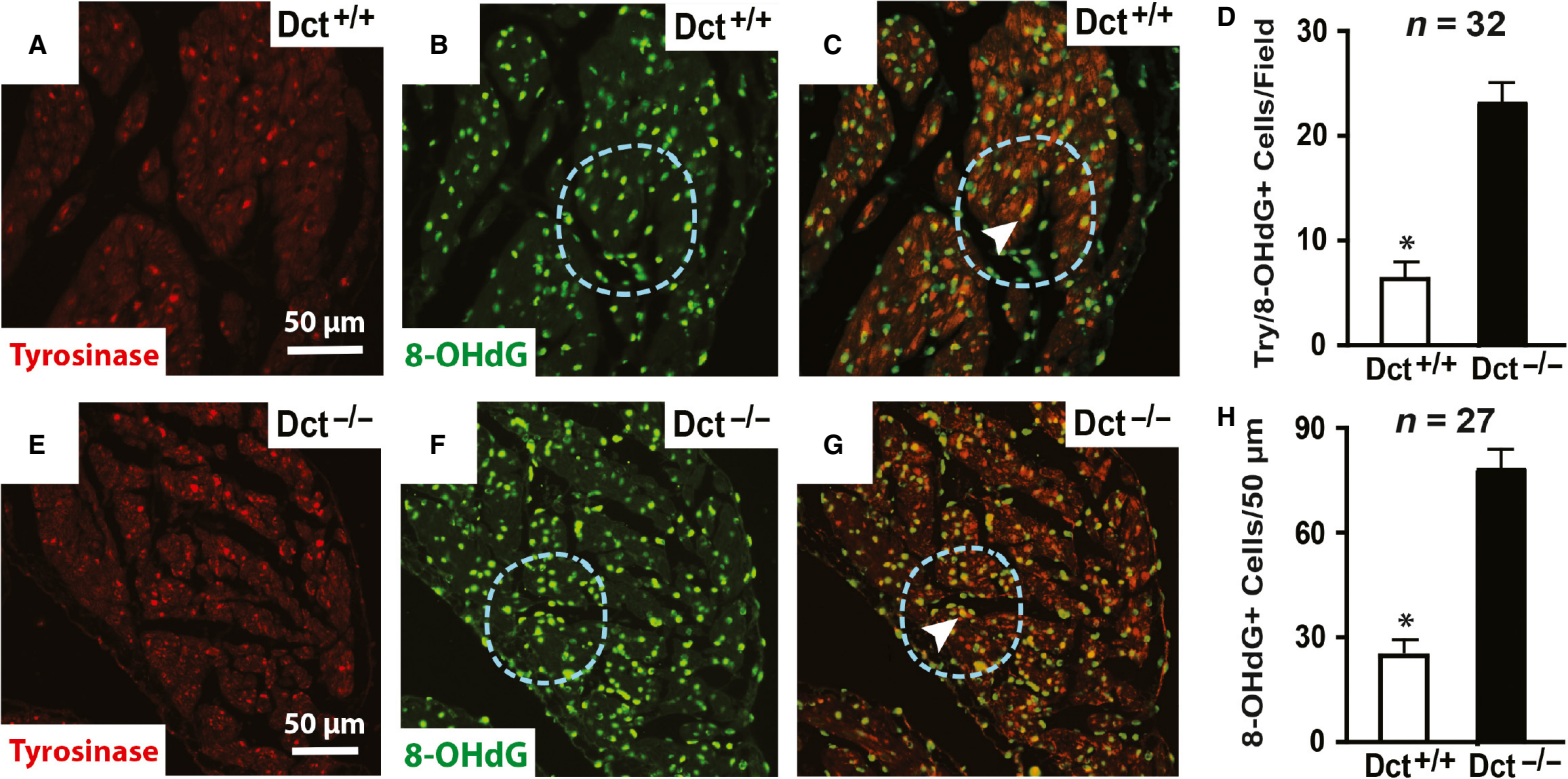
Dct reduces oxidative modifications in CMLCs and the surrounding myocardium. Representative images from wild-type (A–C) and Dct-null (E–G) atria costained with antibodies against tyrosinase (red) and 8-OHdG (green). (A) and (E) show antityrosinase staining, (B) and (F) show anti-8-OHdG staining, and (C) and (G) show merged images using triple filter settings. In (C) and (G), the arrowhead points to CMLCs (yellow) that costain for both tyrosinase (green) and 8-OHdG (red). In (B–C) and (F–G), the blue dashed circle denotes a 50-*μ*m radius surrounding the costained CMLC. The scale bar in (A) applies to (A–C), and scale bar in (E) applies to (E–G). In (D) the bar graph compares the number of cells costained positive for 8-OHdG and tyrosinase per 20× field in Dct-null and wild-type atria. In (H) the bar graph compares the number of anti-8-OHdG stained nuclei within a 50-*μ*m radius of Dct-null and wild-type CMLCs costained positive for tyrosinase and 8-OHdG. At least 27 fields were analyzed from three different hearts in each group. Error bars represent the standard deviation. *P < 0.05.

We previously found that atrial myocytes surrounding CMLCs from Dct-null mice showed evidence of oxidative damage with swollen mitochondria, in contrast to atrial myocytes surrounding CMLCs from wild-type mice (Levin et al. 2009). To determine if Dct-positive CMLCs contribute to the balance of atrial oxidative stress, we examined the atria of Dct-null and wild-type mice for evidence of oxidative modifications using the well-characterized oxidative marker 8-hydro-2′-deoxyguanosine (8-OHdG) (Yamamoto et al. 2003). These experiments showed higher oxidative modification, as assessed by the presence of increased 8-OHdG staining (16.2 ± 4.9% vs. 38.3 ± 5.6%; *P* < 0.001), throughout the atria of Dct-null mice (Fig. 1A–G). To show causality between the lack of Dct and increased oxidative modification, we costained atria from Dct-null and wild-type mice with antibodies against tyrosinase (a specific marker of CMLCs in the heart [Levin et al. 2009]) and 8-OHdG. These studies showed that 8-OHdG colocalizes with tyrosinse-positive CMLCs (Fig. 2), and that the number of 8-OHdG positive atrial myocytes is higher within a 50-*μ*m radius of tyrosinase-positive, Dct-null CMLCs compared to a 50-*μ*m radius surrounding tyrosinase-positive, Dct-positive CMLCs (78.5 ± 8.3 vs. 27.9 ± 5.1; *P* < 0.001).

### Dct in CMLCs reduces cellular superoxide anion expression

**Figure 3:**
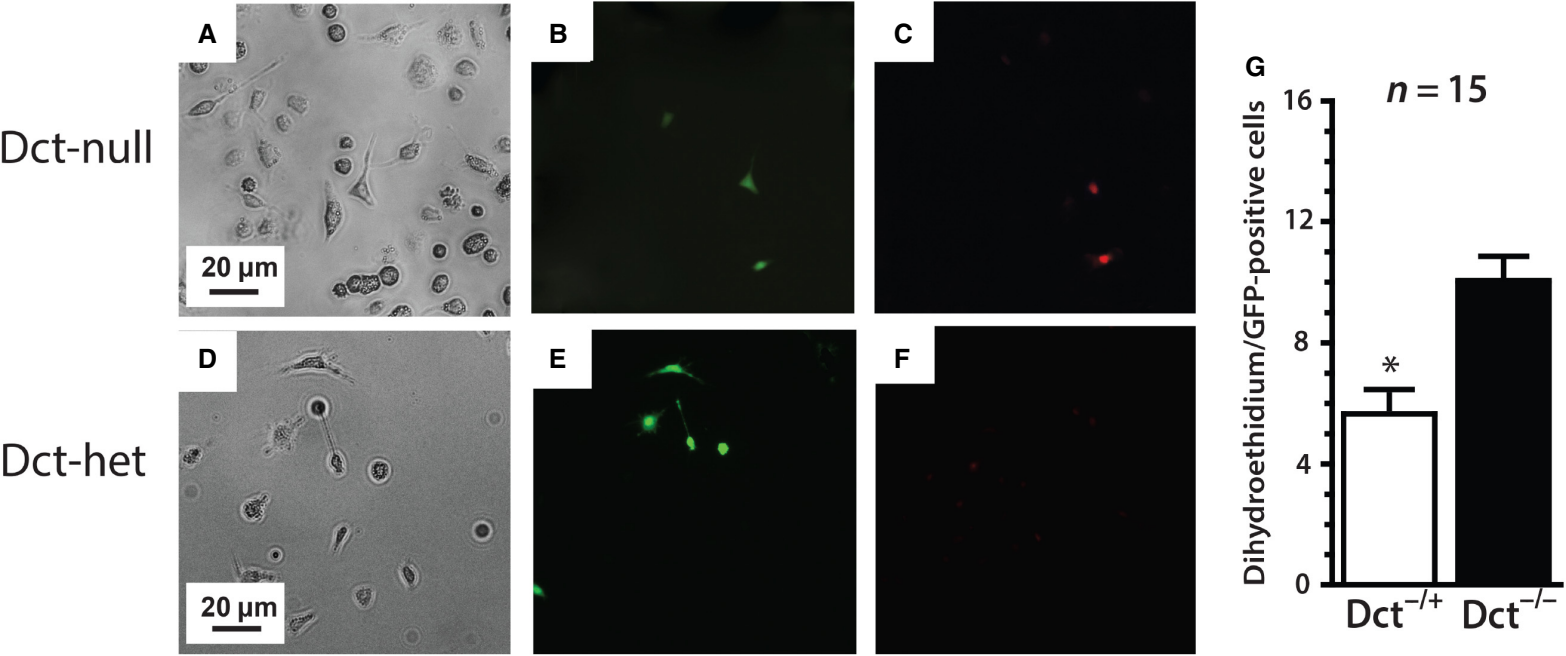
Dct reduces superoxide anion production in CMLCs. (A–C) show representative Dct-null CMLCs and (D–F) show representative Dct-het CMLCs, before and after incubation with 5 μmol/L hydrogen peroxide. (A) and (D) show bright field images, (B) and (E) show GFP fluorescence to identify CMLCs (green) and (C and F) show dihydroethidium fluorescence (red). The scale bar in (A) applies to (A–C), and the scale bar in (D) applies to (D–F). In (G) the bar graph compares relative dihydroethidium fluorescence between Dct-null and Dct-het CMLCs that coexpress red and green fluorescence. At least 15 cells were analyzed in each group from three different hearts. Error bars represent standard deviation, *P < 0.05.

To determine if Dct influences ROS handling in CMLCs, we assessed the amount of superoxide anion produced by individual Dct-null homozygous and heterozygous CMLCs isolated from *DctCre::Z/EG* atria. For these studies we used the specific superoxide marker dihydroethidium to assess the relative amount of superoxide produced by Dct-null and Dct-het CMLCs in response to exogenous hydrogen peroxide. These studies showed that complete loss of Dct increased endogenous superoxide content in CMLCs by ˜60% compared to CMLCs with one functional Dct allele (Fig. 3). These findings further support the hypothesis that Dct in CMLCs contributes to ROS handling. However, it is not clear from these studies if Dct affects superoxide expression by buffering superoxide or affecting its production.

### Dct-null mice have more atrial fibrosis

**Figure 4:**
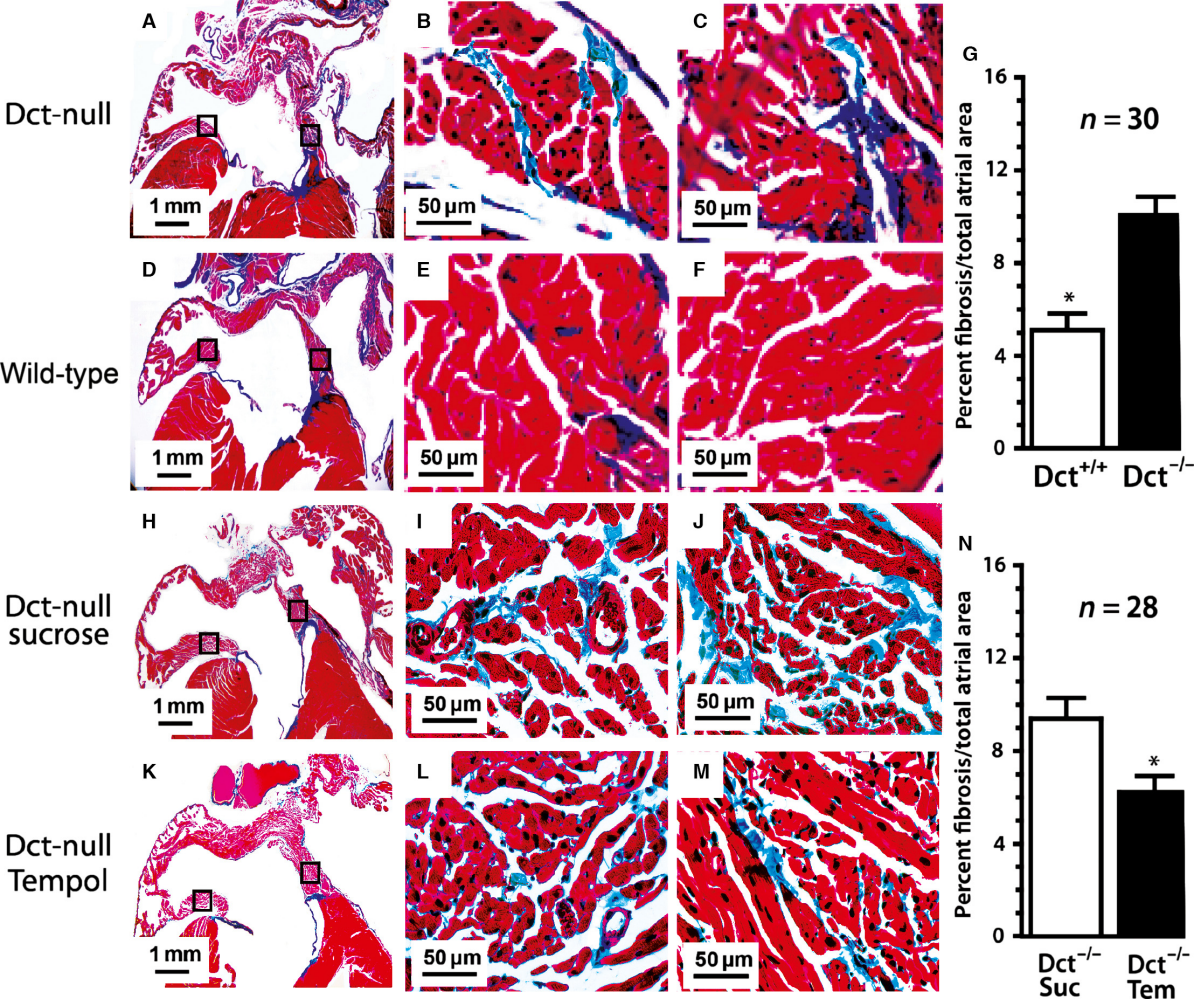
Dct-positive hearts have reduced atrial fibrosis related to atrial ROS. Shown are representative Masson’s trichrome stained hearts from Dct-null mice (A–C), wild-type mice (D–F), Dct-null mice treated with sucrose (H–J) and Dct-null mice treated with the ROS scavenger Tempol (K–M). (A, D, H, and K) are taken at 20× magnification to show overall gross morphology, whereas (B–C, E–F, I–J, and L–M) are taken at 400× to better demonstrate interstitial fibrosis (blue) in each group. The higher power images are taken from the areas demarcated by the black boxes in the lower power images where (B, E, I, and L) are from the intra-atrial septum, and (C, F, J, and M) are from the inferolateral left atrial wall. The bar graph (G) compares the mean percentage of interstitial fibrosis throughout the atrium from Dct-null and wild-type mice. The bar graph (N) compares the mean percentage of interstitial fibrosis in the atrium of Dct-null mice treated with sucrose and Dct-null mice treated with Tempol. At least 28 sections were analyzed from three different hearts in each group. Error bars represent the standard deviation. *P < 0.05.

Since we observed higher 8-OHdG staining in the atria of Dct-null mice compared to wild-type mice, as well as increased superoxide anion in Dct-null CMLCs versus Dct-het CMLCs, we then sought to determine if there is also increased interstitial fibrosis in the atrium of Dct-null mice. Several studies suggest increased oxidative stress induces myocardial interstitial fibrosis (Fukunaga et al. 2012; Lijnen et al. 2006), however, in our initial study we did not see increased myocardial fibrosis in the hearts of Dct-null or Dct-het mice using hematoxylin and eosin staining. To determine if atrial fibrosis was present in the presence or absence of Dct we used the more sensitive trichrome staining technique, which did show increased interstitial fibrosis in the atria of Dct-null mice compared to wild-type atria (10.2 ± 2.4% vs. 5.1 ± 2.0%, *P* < 0.001), as shown in Figure 4A–G. Next, we sought to determine if a connection exists between atrial fibrosis and atrial ROS. For these studies, we treated 4-week-old Dct-null mice with either the ROS scavenger Tempol or sucrose for 6 weeks, and then analyzed the hearts for fibrosis using trichrome staining. These experiments showed that scavenging ROS reduced atrial fibrosis in Dct-null mice, which suggests that increased atrial ROS due to reduced Dct promotes atrial fibrosis, since Dct-null CMLCs produce more ROS (Fig. 4H–N).

### Hydrogen peroxide increases action potential duration in CMLCs

**Figure 5:**
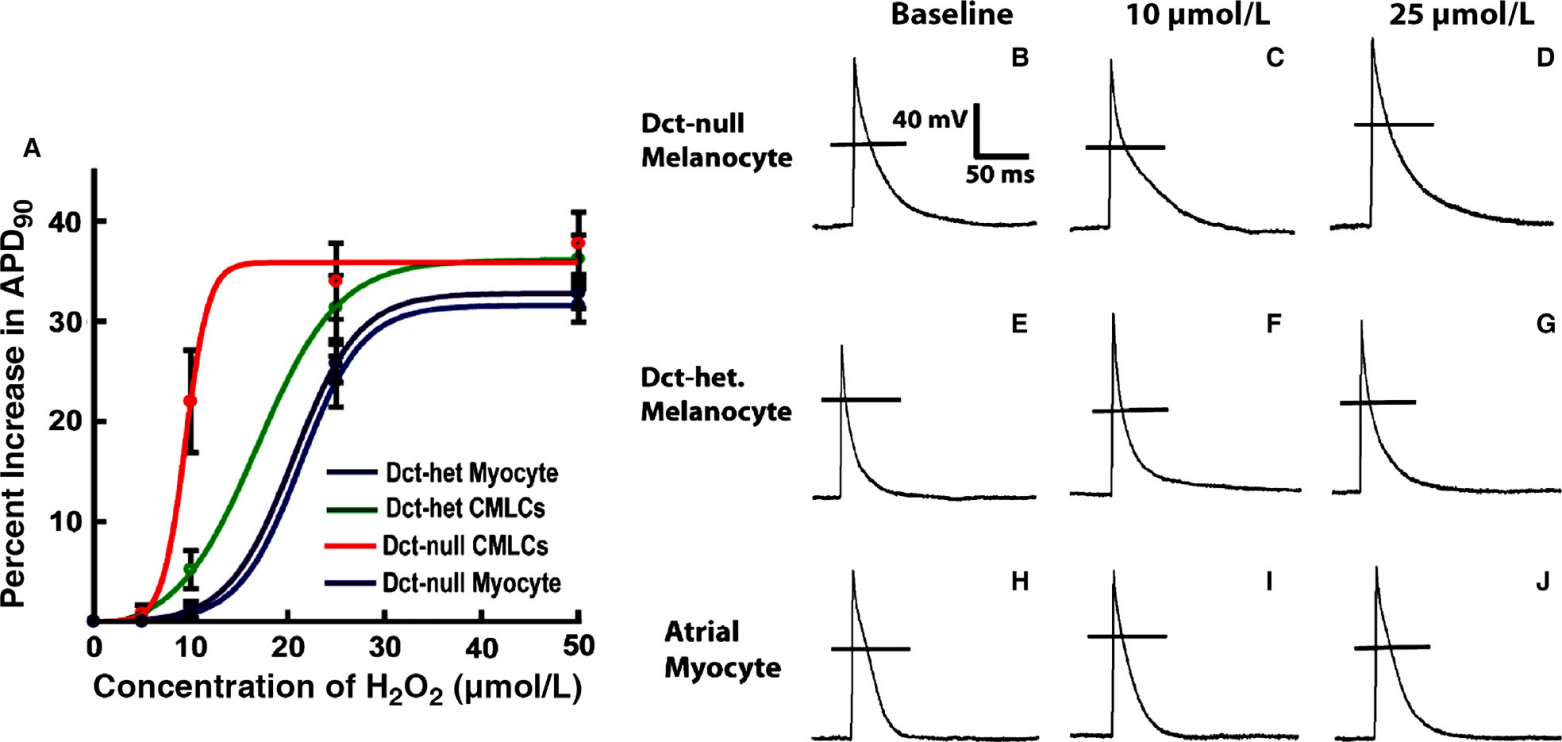
Response of action potential duration in CMLCs and atrial myocytes to hydrogen peroxide. (A) Plotted are the percent increase in action potential duration at 90% repolarization to hydrogen peroxide for Dct-null CMLCs (red), Dct-het CMLCs (green), Dct-het atrial myocytes (blue), and Dct-null atrial myocytes (purple). Data points represent the mean ± standard deviation for at least 11 cells at each concentration of hydrogen peroxide. (B–J) show the electrophysiological response of CMLCs and atrial myocytes to hydrogen peroxide. Displayed are representative action potentials from Dct-null CMLCs (B–D), Dct-het CMLCs (E–G), and Dct-het atrial myocytes (H–J) at baseline (B, E, H), in 10 μmol/L H_2_O_2_ (C, F, I) and in 25 μmol/L H_2_O_2_ (D, G, J). The horizontal line (B–J) denotes the zero potential level.

To assess the electrophysiological response of CMLCs to ROS, we recorded action potentials from CMLCs isolated from Dct-null and Dct-het mice exposed to hydrogen peroxide. These experiments showed action potential duration prolonged in response to increasing concentrations of hydrogen peroxide in both Dct-null and Dct-het CMLCs (Fig. 5; Table 1). However, action potential duration prolonged significantly more in Dct-null CMLCs compared to Dct-het CMLCs in response to the same concentration of hydrogen peroxide (Fig. 5; Table 1). The concentration of hydrogen peroxide required to increase action potential duration to 50% of the maximum action potential duration (EC_50_) was 8.2 ± 1.7 μmol/L for Dct-null CMLCs, compared to 16.8 ± 2.0 μmol/L for Dct-het CMLCs (*P* < 0.001, *n* = 12).

**Table 1:** Action potential parameter response to hydrogen peroxide

	APD_90_ (msec)	APD_50_ (msec)	dV/dt (mV/msec)	V_r_ (mV)
Dct-null CMCLs				
Baseline (*n* = 12)	56.9 ± 7.2*†¶	24.8 ± 8.1*†¶	34.8 ± 9.2	–68.4 ± 6.0
5 μmol/L H_2_O_2_ (*n* = 12)	59.0 ± 8.5*†¶	25.2 ± 7.1*†¶	34.2 ± 8.9	–68.1 ± 6.7
10 μmol/L H_2_O_2_ (*n* = 12)	71.5 ± 13.8*†¶	30.2 ± 8.6*†¶	33.7 ± 9.2	–67.7 ± 7.2
25 μmol/L H_2_O_2_ (*n* = 12)	78.3 ± 12.6*†¶	33.0 ± 9.1*†¶	34.1 ± 9.6	–67.5 ± 6.9
50 μmol/L H_2_O_2_ (*n* = 12)	80.4 ± 11.9*†¶	34.2 ± 7.4*†¶	33.9 ± 9.4	–67.4 ± 7.1
Dct-het. melanocytes				
Baseline (*n* = 12)	32.2 ± 1.5‡	14.3 ± 1.8‡	34.9 ± 9.0	–68.5 ± 7.0
5 μmol/L H_2_O_2_ (*n* = 12)	34.1 ± 1.3‡	14.9 ± 1.4‡	34.6 ± 8.8	–68.0 ± 6.1
10 μmol/L H_2_O_2_ (*n* = 12)	36.8 ± 1.7†‡¶	16.6 ± 1.6‡	34.2 ± 9.4	–67.8 ± 6.8
25 μmol/L H_2_O_2_ (*n* = 12)	45.2 ± 2.8†‡¶	19.2 ± 2.4‡	34.5 ± 9.6	–67.2 ± 7.0
50 μmol/L H_2_O_2_ (*n* = 12)	49.3 ± 3.1†‡¶	20.1 ± 3.2‡	33.8 ± 9.2	–67.3 ± 7.1
Dct-null CMCLs				
Baseline (*n* = 11)	31.3 ± 1.5‡	13.1 ± 1.7‡	36.8 ± 9.8	–68.6 ± 7.2
5 μmol/L H_2_O_2_ (*n* = 11)	32.0 ± 1.7‡	13.3 ± 1.8‡	36.4 ± 10.0	–68.0 ± 7.1
10 μmol/L H_2_O_2_ (*n* = 11)	32.6 ± 1.9*‡	14.9 ± 1.5‡	36.3 ± 8.7	–67.7 ± 6.7
25 μmol/L H_2_O_2_ (*n* = 11)	37.3 ± 2.0‡	18.2 ± 2.3‡	36.8 ± 8.8	–67.4 ± 7.3
50 μmol/L H_2_O_2_ (*n* = 11)	42.4 ± 3.1‡	19.0 ± 3.8‡	35.2 ± 9.3	–67.1 ± 6.8
Dct-het myocytes				
Baseline (*n* = 11)	30.9 ± 1.8‡	12.8 ± 2.0‡	37.1 ± 8.9	–68.3 ± 6.7
5 μmol/L H_2_O_2_ (*n* = 11)	31.7 ± 2.0‡	13.0 ± 2.1‡	36.9 ± 9.3	–68.5 ± 6.9
10 μmol/L H_2_O_2_ (*n* = 11)	32.3 ± 2.2*‡	14.4 ± 1.9‡	36.8 ± 8.8	–68.2 ± 6.4
25 μmol/L H_2_O_2_ (*n* = 11)	37.0 ± 2.5‡	17.6 ± 2.5‡	37.2 ± 9.2	–67.9 ± 7.0
50 μmol/L H_2_O_2_ (*n* = 11)	41.9 ± 2.9‡	18.8 ± 3.6‡	36.6 ± 9.0	–67.4 ± 7.3

* *P* < 0.05 compared to Dct-het. CMCLs at same H_2_O_2_ concentration.

† *P* < 0.05 compared to Dct-het. atrial myocytes at same H_2_O_2_ concentration.

‡ *P* < 0.05 compared to Dct-null CMLCs at same H_2_O_2_ concentration.

¶ *P* < 0.05 compared to Dct-null atrial myocytes at same H_2_O_2_ concentration.

Analogous experiments in atrial myocytes isolated from Dct-null and Dct-het mice also showed action potential duration prolonged in response to increasing concentrations of hydrogen peroxide. These studies showed that the EC_50_ of hydrogen peroxide for increasing action potential duration in Dct-null atrial myocytes was similar to that for Dct-het atrial myocytes (19.9 ± 2.1 μmol/L for Dct-null atrial myocytes and 20.5 ± 1.9 μmol/L for Dct-het atrial myocytes; *P *= NS, *n* = 11). Interestingly, at higher concentrations of hydrogen peroxide (10–50 μmol/L) we found that APD increased more in Dct-het CMLCs compared to that in Dct-het atrial myocytes or Dct-null atrial myocytes (Table 1).

### Hydrogen peroxide triggers more frequent afterdepolarization in CMLCs

**Figure 6:**
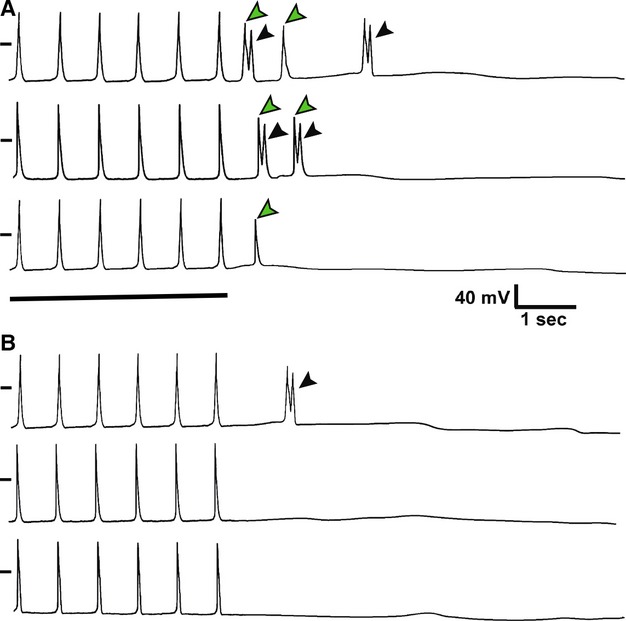
Hydrogen peroxide induces afterdepolarizations in CMLCs. Shown are representative responses of isolated CMLCs and atrial myocytes to a train of current pulses delivered at 1-Hz in (A) 10 μmol/L hydrogen peroxide and (B) at baseline. In both (A) and (B), the top trace is from a Dct-null CMLC, the middle trace is from a Dct-het CMLC and the bottom trace is from a Dct-null atrial myocyte. In the presence of hydrogen peroxide there are increased early afterdepolarizations (black arrowheads) and delayed afterdepolarizations (green arrowheads) in each cell exposed to hydrogen peroxide compared to baseline. The black dash to the left of each trace represents the zero potential mark, and the solid horizontal line denotes the last six pulses of the 1-Hz train in both panels.

To determine if CMLCs may contribute to arrhythmogenic triggers in response to ROS, we induced early and delayed afterdepolarizations in each of the four cell groups (Dct-null CMLCs, Dct-het CMLCs, Dct-null atrial myocytes, and Dct-het atrial myocytes) in response to increasing concentrations of hydrogen peroxide (Fig. 6; Tables2 and 3). Since the response to hydrogen peroxide is the same in both Dct-null and Dct-het atrial myocyte groups, likely because atrial myocytes do not express Dct, we present these data together to simply Figure 6, Tables2 and 3. Interestingly, we found that both early and delayed afterdepolarizations were elicited more frequently in CMLCs than atrial myocytes at lower concentrations of hydrogen peroxide (5 and 10 μmol/L; Tables2 and 3). In addition, we saw more delayed afterdepolarizations in Dct-null CMLCs compared to either Dct-het CMLCs or atrial myocytes, also at lower hydrogen peroxide concentrations (5 and 10 μmol/L; Table 2). On the other hand, the frequency of induced early afterdepolarizations was not significantly different between Dct-null and Dct-het CMLCs in response to hydrogen peroxide at a concentration of 25–50 μmol/L (Table 3). These findings lend further support for the hypothesis that CMLCs are more sensitive to ROS-induced electrophysiological remodeling, with the potential for initiating arrhythmogenic triggers, compared to atrial myocytes.

**Table 2:** Mean number of induced delayed afterdepolarizations per cell

	Dct-null CMLCs *N* = 11	Dct-het CMLCs *N* = 12	Atrial myocytes *N* = 23
Baseline	0.83 ± 0.22*†	0[Table-fn tf2-4]	0[Table-fn tf2-4]
5 μmol/L H_2_O_2_	1.49 ± 0.34*†	0.88 ± 0.26†‡	0.32 ± 0.25*‡
10 μmol/L H_2_O_2_	1.67 ± 0.32*†	1.04 ± 0.28†‡	0.44 ± 0.30*‡
25 μmol/L H_2_O_2_	1.92 ± 0.41†	1.22 ± 0.42	0.72 ± 0.38‡
50 μmol/L H_2_O_2_	2.15 ± 0.40†	1.64 ± 0.42	1.09 ± 0.40‡

Atrial myocyte group contains data combined from both Dct-null and Dct-het atrial myocytes.

* *P* < 0.05 compared to Dct-het melanocytes at same H_2_O_2_ concentration.

† *P* < 0.05 compared to Dct-null and Dct-het atrial myocytes at same H_2_O_2_ concentration.

‡ *P* < 0.05 compared to Dct-null melanocytes at same H_2_O_2_ concentration.

**Table 3:** Mean number of induced early afterdepolarizations per cell

	Dct-null CMLCs *N* = 11	Dct-het CMLCs *N* = 12	Atrial myocytes *N* = 23
Baseline	0.89 ± 0.34*†	0‡	0‡
5 μmol/L H_2_O_2_	1.61 ± 0.32*†	0.87 ± 0.23†‡	0.41 ± 0.21*‡
10 μmol/L H_2_O_2_	2.22 ± 0.30*†	1.54 ± 0.28†‡	0.86 ± 0.27*‡
25 μmol/L H_2_O_2_	2.98 ± 0.34†	2.46 ± 0.32†	1.39 ± 0.35*‡
50 μmol/L H_2_O_2_	3.12 ± 0.35†	2.78 ± 0.37†	1.64 ± 0.39*‡

Atrial myocyte group contains data combined from both Dct-null and Dct-het atrial myocytes.

* *P* < 0.05 compared to Dct-het melanocytes at same H_2_O_2_ concentration.

† *P* < 0.05 compared to Dct-null and Dct-het atrial myocytes at same H_2_O_2_ concentration.

‡ *P* < 0.05 compared to Dct-null melanocytes at same H_2_O_2_ concentration.

## Discussion

Recent studies have uncovered several unique cell populations in the atrium and pulmonary veins that may contribute to atrial fibrillation in patients (Levin et al. 2009; Perez-Lugones et al. 2003; Gherghiceanu et al. 2008). However, just identifying a novel cell population in the heart does not help to understand its native role or contribution to arrhythmogenesis. In the clinical setting there are specific conditions which trigger atrial fibrillation. If connections between defined stimuli that initiate AF and a unique cell population can be established, then this may lead to a better understanding of the mechanisms underlying AF and provide guidance for improving therapies. Along these lines, evidence supports the role of oxidative stress in the promotion of atrial fibrillation (Kim et al. 2005) by inducing interstitial fibrosis (Van Wagoner 2008) and/or ion channel modification (Wang et al. 2007). The studies described in this report suggest CMLCs contribute to the genesis of atrial arrhythmias by affecting the balance of reactive oxygen species and influencing oxidative stress throughout the atrium, which then contributes to atrial remodeling.

Melanin synthesis enzymes evolved as a pathway to scavenge and reduce ROS. While most reactive oxygen species are reduced by melanin in the skin, the enzymes involved with melanin synthesis are also capable of independently reducing ROS (Perluigi et al. 2003; Jiang et al. 2010). This is particularly important with regards to the role CMLCs may play in clinical atrial arrhythmias, where the human analogues of these cells express both tyrosinase and dopachrome tautomerase but do not produce eumelanin (Levin et al. 2009). Therefore, it seems CMLCs may be present in the heart to help maintain the balance of atrial ROS. In addition, it appears that just the expression of melanin synthesis enzymes in CMLCs, independent of melanin, contributes to the balance of atrial ROS. The fact that Dct-null CMLCs have increased superoxide and action potential duration in response to lower concentrations of hydrogen peroxide, compared to Dct-het CMLCs or atrial myocytes, suggests that Dct plays a role in maintaining the balance of ROS and electrical function in CMLCs. Interestingly, we also found that Dct-null mice have evidence of increased oxidative modification and fibrosis throughout their atria compared to wild-type mice, which could be prevented by treating Dct-null mice with ROS scavengers. Furthermore, we previously reported that ROS scavengers reduced the incidence of inducible atrial arrhythmias in Dct-null mice (Levin et al. 2009). Together these findings suggest that Dct in CMLCs is involved with regulating oxidative stress throughout the atrium that contributes to atrial structural remodeling and arrhythmias.

In addition to ROS, alterations in intracellular calcium can also induce structural and electrical remodeling in the atrium that promotes AF (Nattel and Dobrev 2012). We previously showed that CMLCs from Dct-null mice have alterations in intracellular calcium that promotes delayed afterdepolarizations. In this study, we show that exogenous ROS induces more delayed afterdepolarizations in Dct-null CMLCs compared to Dct-het CMLCs or atrial myocytes. While we did not directly investigate the effects of ROS or Dct on intracellular calcium handling in CMLCs, there is currently no evidence that supports a direct interaction between Dct and calcium, Instead, it is more likely that increased ROS in the absence of Dct in CMLCs induces oxidative modifications of calcium handling proteins that indirectly lead to altered intracellular calcium handling in these cells. None the less, changes in intracellular calcium handling in Dct-null CMLCs probably does contribute to atrial remodeling that promotes atrial arrhythmias, even if it is due to an indirect effect of increased ROS.

For technical reasons, to conduct studies in live cells we compared Dct-null CMLCs to Dct-het CMLCs, instead of wild-type CMLCs. This was necessary since we had to have at least one Cre allele present in live CMLCs to express GFP from the Z/EG allele and fluorescently label them. To validate the use of Dct-het cells as a control group we need to know that Dct-het CMLCs and wild-type CMLCs have similar Dct enzymatic activity. While we do not have exact measurements of Dct enzymatic activity from Dct –/+ and Dct +/+ CMLCs, if anything, the enzymatic activity of Dct is likely to be lower in Dct –/+ CMLCs compared to wild-type CMLCs. Therefore, this should reduce the differences we would expect to see between Dct –/+ and Dct –/– cells compared to wild-type control cells. The fact that we are able to measure significantly lower superoxide anion levels in Dct –/+ compared to Dct –/– CMLCs, as well as increased action potential duration in response to hydrogen peroxide in Dct –/– versus Dct –/+ CMLCs, suggests that using Dct –/+ CMLCs is an acceptable control for these experiments.

To date, CMLCs are the only cells known that express melanin synthesis enzymes in the atrium and this feature distinguishes them from cardiomyocytes and other normal atrial cell populations. The fact that electrical remodeling and afterdepolarizations are more readily induced in CMLCs by lower concentrations of hydrogen peroxide suggests these cells have less robust ROS scavenging pathways than atrial myocytes. Based upon these differences, further work may show that CMLCs could be employed as therapeutic targets for atrial fibrillation to modulate atrial oxidative stress.

## Conclusions

Dopachrome tautomerase (Dct) expressed by CMLCs influences the balance of reactive oxygen species through the atrium. The absence of Dct renders individual CMLCs more susceptible to triggering afterdepolarizations and increases atrial fibrosis that can be prevented by ROS scavengers.
